# Serum-derived piR-hsa-164586 of extracellular vesicles as a novel biomarker for early diagnosis of non-small cell lung cancer

**DOI:** 10.3389/fonc.2022.850363

**Published:** 2022-09-28

**Authors:** Yanli Li, Yanhan Dong, Shupeng Zhao, Jinning Gao, Xiaodan Hao, Zibo Wang, Meng Li, Mengyuan Wang, Yiming Liu, Xiaoling Yu, Wenhua Xu

**Affiliations:** ^1^ Department of Pathology and Pathophysiology, The Medical Faculty of Qingdao University, Qingdao, China; ^2^ Institute of Translational Medicine, Qingdao University, Qingdao, China; ^3^ Asset and Laboratory Management Office, Qingdao University, Qingdao, China; ^4^ Department of Inspection, The Medical Faculty of Qingdao University, Qingdao, China

**Keywords:** non-small cell lung cancer, serum, extracellular vesicles, piRNA, liquid biopsy, early diagnosis, cancer

## Abstract

Non-small cell lung cancer (NSCLC) is a major cause of death in those with malignant tumors. To achieve the early diagnosis of NSCLC, we investigated serum-derived Piwi-interacting RNA (piRNA) of extracellular vesicles to filter diagnostic biomarkers for NSCLC. High-throughput sequencing from cancerous tissues and adjacent noncancerous tissues in patients with NSCLC was first applied to recognize candidate piRNAs as diagnostic biomarkers. These screened piRNAs were further validated in 115 patients (including 95 cases in stage I) and 47 healthy individuals using quantitative real-time PCR (qRT-PCR). We showed that piR-hsa-164586 was significantly upregulated compared with paracancerous tissues and extracellular vesicles from the serum samples of healthy individuals. Moreover, the area under the curve (AUC) value of piR-hsa-164586 was 0.623 and 0.624 to distinguish patients with all stages or stage I of NSCLC, respectively, from healthy individuals. The diagnostic performance of piR-hsa-164586 was greatly improved compared with the cytokeratin-19-fragment (CYFRA21-1). Additionally, piR-hs-164586 was associated with the clinical characteristics of patients with NSCLC. Its expression was associated with the age and TNM stage of patients with NSCLC, indicating that it can serve as an effective and promising biomarker for the early diagnosis of NSCLC.

## Introduction

Lung cancer is the leading cause of cancer-related mortality worldwide (1, 2). Non-small cell lung cancer (NSCLC) accounts for about 80%–85% of all lung cancers (3). Squamous cell carcinoma (SC) (4) and adenocarcinoma (AC) (5) are the two main pathological types of NSCLC (6), and AC is the most frequent type of NSCLC (7). Most patients are already at an advanced stage when they are diagnosed, and the 5-year survival rate is very unsatisfactory, the global five-year survival rate is only about 5% (8, 9). Due to the lack of assessment of prognostic factors, patients may not receive more optimal treatment, which increases early mortality. However, biomarkers can solve these problems, since genetic changes may precede obvious histopathological changes. Owing to the lack of effective diagnostic biomarkers, histopathological biopsy technique is still the most accurate method for the diagnosis of NSCLC (10). However, this method is invasive and may cause a series of complications, including tissue and blood vessel injury, hemorrhage, and tumor cell implantation and metastasis (11–14). Existing clinical protein-type tumor markers are not sensitive and specific enough (15), so there is an urgent need to discover new tumor markers to compensate for the shortcomings of existing methods and further improve the diagnostic performance of the existing tumor markers. Exosomes are nanovesicles secreted by cells, and they contain lipids, proteins, and nucleic acids (16). After they are released into the extracellular environment, they can be detected in blood, urine, and saliva (17). Some molecules, such as proteins and piRNA loaded by extracellular vesicles, can stably exist in the circulatory system and participate in cell-to-cell communication (18). The emerging roles of genes in cancer diagnosis are played by piwi-interacting RNAs (piRNAs), a novel class of small non-coding RNAs (sncRNAs) with a length of 24–31 nucleotides (nt) that bind to the PIWI protein family and play a regulatory role at the gene level (19), such as silencing transcriptional genes, maintaining germline and stem cell performance,and regulating translation and mRNA stability. Previous studies have shown that piRNAs are dysregulated in different types of cancer, and some of them are tumor-specific (20). Recent studies have shown that piRNAs in extracellular vesicles are not only stable in circulation but can also be effective biomarkers for cancer diagnosis and screening (21). However, it remains unknown whether piRNA derived from serum extracellular vesicles can be used as a clinical biomarker for NSCLC.

In this study, we conducted a series of experiments to explore novel serum-derived piRNAs of extracellular vesicles as biomarkers for the diagnosis of NSCLC. First, differently expressed and upregulated piRNAs were screened out from the sequencing results of cancerous and paracancerous tissues of patients with NSCLC. Then, we verified them in serum-derived extracellular vesicles and constructed receiver operating characteristic (ROC) curves to analyze the diagnostic effects of piRNAs in extracellular vesicles. Finally, we assessed their performance as an indicator of the postoperative prognosis of the patients and correlated their expression level with the clinical characteristics of patients with NSCLC. Our research showed that piR-hsa-164586 of extracellular vesicles can be used as a novel biomarker for diagnosing NSCLC.

## Materials and methods

### Subjects

From March 2021 to September 2021, 162 individuals were enrolled at the Affiliated Hospital of Qingdao University, including 115 patients with NSCLC and 47 healthy individuals. All of the patients in this study received a diagnosis of NSCLC by histopathological examination, and tumor staging was determined in accordance with the Eighth Edition American Joint Committee on Cancer (AJCC) Cancer Staging Manual (22). None of the patients had received radiotherapy or chemotherapy before peripheral venous blood collection, and they had no other diseases. Furthermore, 29 pairs of serum samples from patients with NSCLC before and one week after surgery (13 men and 16 women, mean age of 61.0 years) were collected. All of the samples were collected with the informed consent of the patients, and this study was approved by the Ethics Committee of Qingdao University Medical Department.

### Isolation and identification of extracellular vesicles

Exosomes were extracted and isolated by the Hieff™ Quick extracellular vesicle isolation kit (for serum/plasma) (Yeasen Biotechnology, Shanghai, China) from each 1-ml serum sample. Specifically, each serum was centrifuged at 10,000×g for 20 min at 4°C to remove cell debris, and then centrifuged at 10,000×g for 60 min at 4°C to collect extracellular vesicles. Finally, extracellular vesicles were resuspended in Phosphate Buffered Saline (PBS) (Coolaber, Shanghai, China) for subsequent analysis. Exosomes were identified in three experiments.

### Transmission electron microscope analysis

The morphology of extracellular vesicles was observed through TEM. First, 5 µl of extracellular vesicles suspension was added to the Formvar-carbon sample loaded with copper net; then, we placed the copper mesh on 50 µl of 1% glutaraldehyde for 5 min; finally, negative staining was performed, and electron microscope images were taken at 80 kV.

### Nanoparticle-tracking analysis

The particle size and concentration of extracellular vesicles were measured by using nanoparticle-tracking analysis (NTA) at Viva Cell Biosciences with ZetaView PMX 110 (Particle Metrix, Meerbusch, Germany) and the corresponding Zeta View 8.04.02 software. The isolated samples of extracellular vesicles were appropriately diluted with 1× PBS buffer (Biological Industries, Shanghai, China) to measure the particle size and concentration. NTA measurements were recorded and analyzed at 11 positions. The Zeta View system was calibrated using 110 nm polystyrene particles. The temperature was maintained around 23°C and 30°C.

### Western blot analysis

A suspension of extracellular vesicles (400 µl) was prepared by using RIPA Lysis Buffer with 1% PMSF (Meilun, Dalian, China). Then, 80 ng of extracted protein from extracellular vesicles was separated by 10% SDS-PAGE and transferred to PVDF Western blotting membranes (Sigma-aldrich,Mannheim,Germany). After blocking with 5% skim milk for 2 h, the membranes were incubated with primary antibodies at 4°C overnight and then incubated with secondary antibodies (1:8,000, Abclonal, Wuhan, China) for 2 h at room temperature. We used a protein imager (Amersham, Boston, US), to visualize protein bands. Primary antibodies included CD9 (1:1,000, Abcam, ab254175, MA, Cambridge, UK), CD81 (1:1,000, Abcam, ab109201, MA, Cambridge, UK), and TSG101 (1:1,000, Abcam, ab83, MA, Cambridge, UK).

### RNA extraction, reverse transcription, and real-time quantitative PCR

Each 400 µl serum extracellular vesicle suspension was prepared for RNA extraction as described in what follows. Exosomal RNA was extracted using TRIZOL reagent (Takara, Dalian, China), and reverse transcription was performed with an miRNA 1st Strand cDNA Synthesis Kit (Nanjing Novozan Biotechnology, Nanjing, China). The quality and purity of each sample isolated from extracellular vesicles were measured using a Bio-DROP spectrophotometer (Bio-Tek, Vermont, US) and agarose gel electrophoresis (**Figures S1A, B**). qRT-PCR was performed using miRNA Universal SYBR qPCR Master Mix (Nanjing Novozan Biotechnology, Nanjing, China). Reverse transcription was performed on a T100TM Thermal Cycler (Bio-Rad, California, US). qRT-PCR analysis was performed on a CFX Connect TM Real-Time System (Bio-Rad, California, USA). The relative expression level of genes was evaluated using the 2^−△CT^ [2^-(CtpiRNA-CtU6)^] method (23). U6 was used as an internal control (18). Each sample was analyzed in triplicate. The primers used in the experiment are shown in **Table 1**.

**Table 1:** qPCR primer sequence.

Gene	Primer sequence
piR-hsa-164586	F: 5’°- CGCGTGAGAACTGAATTCCATA -3’
	R: S’- AGTGCAGGGTCCGAGGTATT -3’
piR-hsa-93750	F: S’- CGCGTTACTTGATGACAATAAAATA -3’
	R: S’- AGTGCAGGGTCCGAGGTATT -3’
piR-hsa-137463	F: 5’-CGCGTAGCTTATCAGACTGATG-3’
	R: S’°-AGTGCAGGGTCCGAGGTATT-3’
piR-hsa-34316	F: S'-CGCGTTACTTGATGACAATAAAATAT-3’
	R: 5S’°- AGTGCAGGGTCCGAGGTATT-3’
piR-hsa-148318	F: S’- CGCGTAGCTTATCAGACTGATG -3’
	R: 5S’°-AGTGCAGGGTCCGAGGTATT -3’
piR-hsa-171929	F: S’- CGCGTAGCTTATCAGACTGATG -3’
	R: 5S’°-AGTGCAGGGTCCGAGGTATT -3’
piR-hsa-158651	F: 5’°- CGCGTGAGAACTGAATTCCATA -3’
	R: 5S’°-AGTGCAGGGTCCGAGGTATT -3’
piR-hsa-143056	F: 5’°- ATGATGAATGCCAACCGCT-3’
	R: S’-AGTGCAGGGTCCGAGGTATT -3’
U6	F:5’-CTCGCTTCGGCAGCACA-3’
	R: S’-AACGCTTCACGAATTTGCGT-3’

F represents forward: R represents reverse.

### CYFRA21-1 measurement

For measuring serum CYFRA21-1 concentration, we used a chemiluminescence method. The range of the reference values of serum CYFRA21-1 was 0–3.3 ng/ml.

### Statistical analysis

GraphPad Prism 8.0 (GraphPad Software, San Diego, CA, USA) and SPSS 26.0 (IBM, Eningen, Germany) were used for statistical analysis. The Kolmogorov–Smirnov test was used to evaluate the normality of the distribution. The t-test or one-way ANOVA was performed on those values conforming to a normal distribution, while those that did not conform to a normal distribution were compared using the Mann–Whitney rank-sum test. The ROC curve and the corresponding AUC were obtained using a logistic regression analysis model combined with pathological diagnosis to determine the cutoff value. The results of the numerical representation methods were expressed as the mean ± standard deviation. All tests were two-tailed, and p <0.05 was considered statistically significant.

## Results

### Exosomal piR-hsa-164586 is significantly upregulated in NSCLC patients

**Figure 1:**
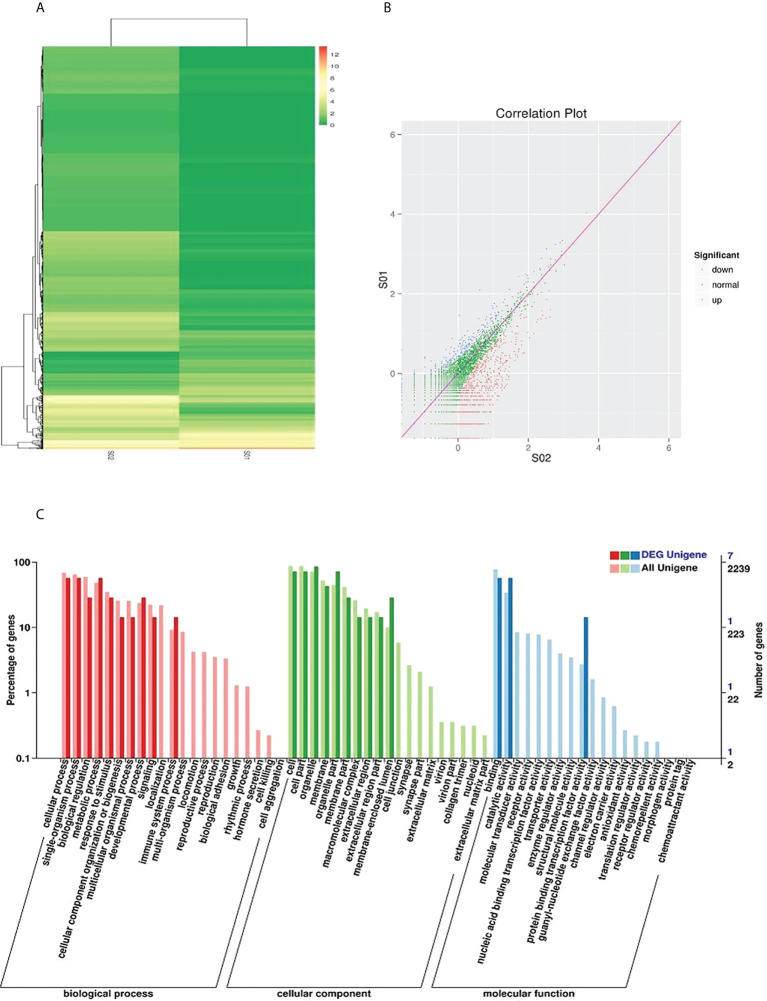
Sequencing analysis of piRNAs in cancerous and adjacent noncancerous tissues of NSCLC. **(A)** Hierarchical cluster and **(B)** Correlation Plot analysis of differential piRNAs betweencarcinoma tissues and para-carcinoma tissues of NSCLC patients **(C)** GO analysis (SOl, para-carcinoma tissues; SO2, carcinoma tissues).

To identify piRNAs with imbalanced expression, we performed high-throughput sequencing on cancerous and adjacent tissues from NSCLC patients and performed microarray analysis (**Figures 1A, B**). As shown in **Figure S2A** and **Table S1**, we found that eight types of piRNAs were significantly upregulated. These eight most differentially expressed piRNAs were further verified to be regulated in serum extracellular vesicles by qRT-PCR. The upregulation of piR-hsa-164586 expression levels was statistically significant was found between patients and healthy individuals (**Figures S2B–I**). The Gene Ontology (GO) enrichment analysis identified the function of these differentially regulated genes (**Figure 1C**). Among these genes, binding was the most common relevant molecular function.

### Isolation of extracellular vesicles from clinical serum

**Figure 2:**
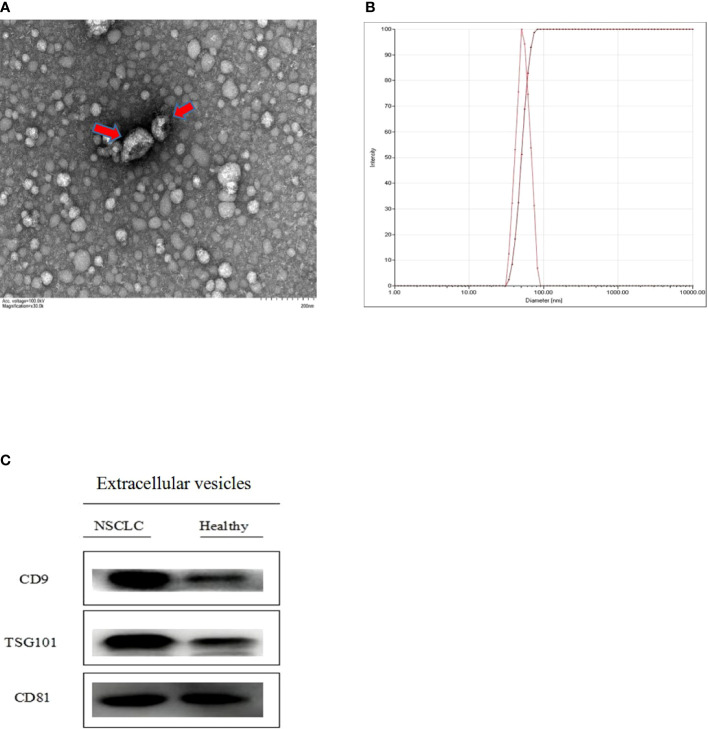
Identification of extracellular vesicles isolated from serum **(A)** the morphological characteristics of extracellular vesicles was observed by transmission electron microscope (TEM) **(B)** the diameter distribution and intensity of extracellular vesicles were analyzed through nano particle tracking analysis (NTA) **(C)** Extracellular vesicles specitic marker proteins (CD9, CD81 and TSG101) were detected by Western blotting.

Isolated extracellular vesicles from the serum of patients with NSCLC and healthy individuals were analyzed by TEM, NTA, and Western blotting. As shown in **Figures 2A, B**, extracellular vesicles presented a typical tea tray-like structure, and the measured particle sizes were concentrated between 50 and 100 nm. The specific marker proteins (CD9, CD81, and TSG101) were also detected and enriched in the extracellular vesicles (**Figure 2C**).

### Verification of the stability of piRNAs from extracellular vesicle of serum

**Figure 3:**
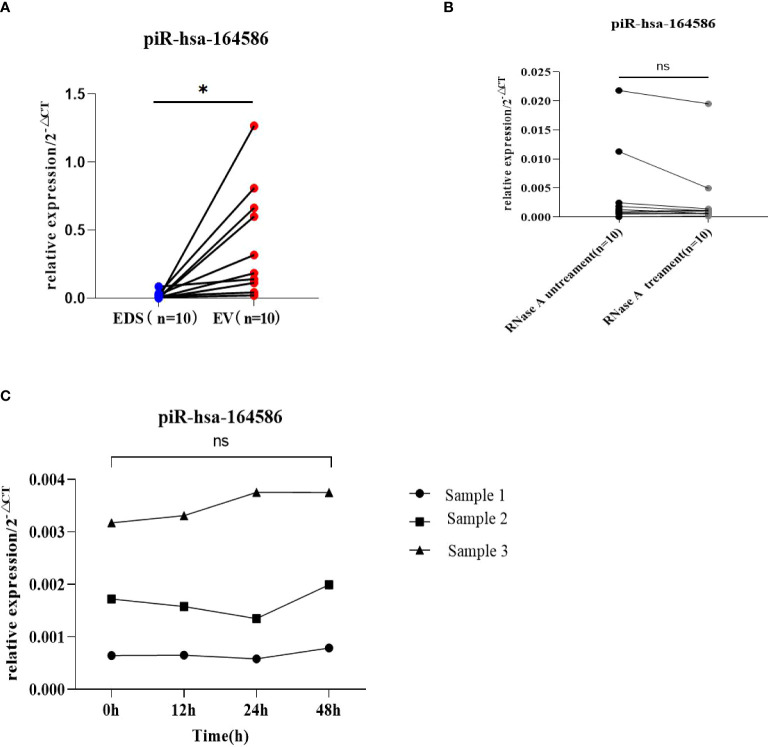
Evaluation of the stability of piR-164586 of extracellular vesicle in serum samples **(A)** Expression level of piR-hsa-164586 in extracellular vesicles (EV) and extracellular vesicles depleted suspension (EDS); **(B)** qRT-PCR analysis of the expression level of piR-hsa-164586 in extracellular vesicles with or without RNase A treatment; **(C)** Expression level of piR-hsa-164586 of extracellular vesicles stored at room temperature for different times (0h, 12h, 24h and 48h) (ns, no significance; *p≤0.05).

In clinical application, the stability of tumor markers is the key factor in tumor screening. Here, we verified the stability of piR-has-164586 from extracellular vesicles of serum samples. First, we tested whether piR-hsa-164586 was enriched in serum extracellular vesicles. We separately detected the expression levels of piRNA in extracellular vesicles and extracellular vesicle-depleted suspension (EDS). We found that the expression levels of piRNAs in extracellular vesicles were much higher than those in EDS (p <0.05; **Figure 3A**). Subsequently, the suspensions of extracellular vesicles were treated with RNase A, but this treatment did not have a significant effect on piRNA expression of extracellular vesicles (p >0.05; **Figure 3B**). After storing the suspension of extracellular vesicles at room temperature for different time periods (0 h, 12 h, 24 h, and 48 h), the expression of piRNA was measured. As shown in **Figure 3C**, the expression of piRNA from extracellular vesicles did not change with time (p >0.05).

### Diagnostic efficacy of piRNAs from serum extracellular vesicles for NSCLC

**Figure 4:**
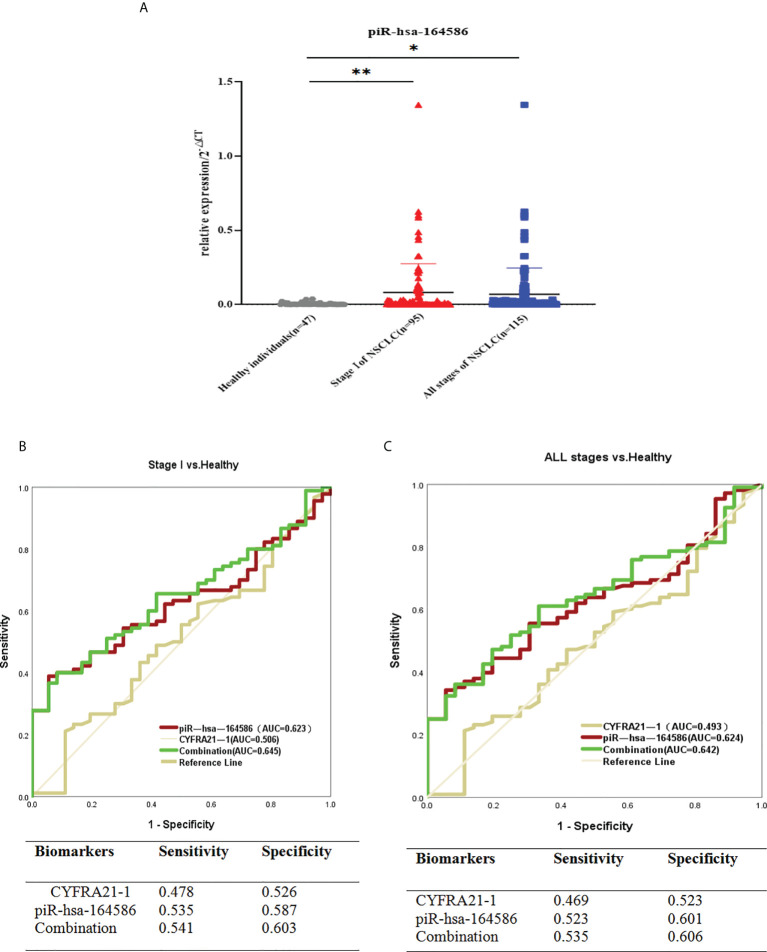
Serum-derived piR-hsa-164586 of extracellular vesicles is a potential biomarker for NSCLC patients. **(A)** qRT-PCR analysis the expression level of piR-hsa-164586 of extracellular vesicles in patients with stage of NSCLC (n = 95), all stage of NSCLC (n = 115) and healthy persons (n = 47). (*p < 0.05,**p < 0.01); **(B, C)** ROC curve analysis for piR-hsa-164586 of extracellular vesicles in distinguishing stageor all stage of NSCLC from healthy individuals.

In addition, the expression level of piR-hsa-164586 in extracellular vesicles taken from serum samples was significantly upregulated in patients with NSCLC compared with healthy individuals (**Figure 4A**).

To evaluate the diagnostic efficacy of piR-hsa-164586 from extracellular vesicles on NSCLC, the ROC curves were drawn for analysis. Given that serum biomarker CYFRA21-1 is widely used in clinical practice as a control (24), we evaluated the diagnostic value of CYFRA21-1 and piR-hsa-164586 of extracellular vesicles for stage I NSCLC patients. As shown in **Figure 4B**, the AUCs of CYFRA21-1 and piR-has-164586 were 0.506 (95% confidence interval [CI], 0.395–0.617) and 0.623 (95% CI, 0.527–0.720), respectively. The AUC of the combination of piR-hsa-164586 and CYFRA21-1 was 0.645 (95% CI, 0.550–0.741), with a sensitivity of 0.541 and a specificity of 0.603.

Likewise, when all stages of NSCLC were compared with healthy individuals, the AUC of piR-hsa-164586 was 0.624 (95% CI, 0.530–0.718), with a sensitivity of 0.523 and a specificity of 0.601. For the combination of piR-hsa-164586 and CYFRA21-1, the AUC was 0.642 (95% CI, 0.550–0.734), with a sensitivity of 0.535 and a specificity of 0.606 (**Figure 4C**).

### Evaluation of piR-hsa-164586 of extracellular vesicles from serum on postoperative prognostic performance

**Figure 5:**
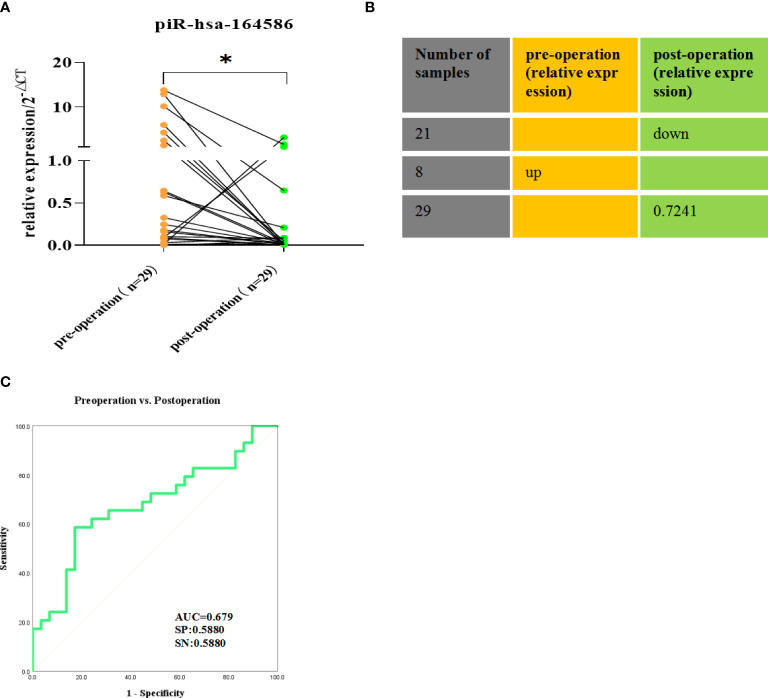
Comparison of the expression of piR-hsa-164586 of extracellular vesicles in preoperative and postoperative serum samples of NSCLC patients.(ns, no significance; *p < 0.05). **(A)** The relative expression of piR-hsa-164586 of extracellular vesicles before and after operation; **(B)** The percentage of decreased expression of piR-hsa-164586 in postoperative samples is 72.41% **(C)** ROC curve analysis of postoperative prognostic performance of NSCLC patients(SN, sensitivity; SP, specificity).

Serum samples from 29 patients were taken before surgery and one week after surgery. The results showed that the expression level of piR-hsa-164586 after surgery was significantly lower than that before surgery (**Figure 5A**, p <0.05). in particular, those with high expression showed a significant downward trend, and 72.41% of the 29 patients showed a downward trend (**Figure 5B**). The ROC curve was used to evaluate the diagnostic performance of piR-hsa-164586 for the postoperative prognosis of patients, and the AUC was 0.679, with a sensitivity of 0.588 and a specificity of 0.588 (**Figure 5C**).

### Correlation between piRNAs of extracellular vesicles from serum and clinical characteristics of patients with NSCLC

Given that the intention of this study was to examine patients who had not received any chemotherapy or radiotherapy, patients in stage IV were not included in this study. Based on the TNM stage, we divided the serum samples into stages I–III. As shown in **Figure S3A**, the expression level of piRNAs of extracellular vesicles in the serum of cancer stage I patients was significantly higher than that of the healthy group, whereas that in stage III and IV patients did not show this trend. When the three groups were compared, it became clear that the expression of piR-hsa-164586 was correlated with TNM (**Table 2**). Lymph node metastasis is also critical to cancer progression (25). As shown in **Figure S3B**, the expression of piRNAs in patients without lymph node metastasis was dramatically higher than that in healthy controls, but there was no such difference in patients with lymph node metastasis.

**Table 2:** Correlation between piR-hsa-164586 levels (2^ΔCT^) and clinical characteristics of patients with NSCLC (Mean+Standard Deviation).

Categories	Case	piR-hsa-164586	p-value
Age (years)			
<55	35	0.0336+0.0968	0.0310*
56-65	48	0.1014+0.2383	
>66	32	0.0606+0.1251	
Gender			
Male	28	0.0168+0.0322	0.0697
Female	87	0.0864+0.1999	
NSCLC			
Subtype			
AC	109	0.0726+0.1812	0.5845
SCC	6	0.011940.0100	
Tumor Stage			
I	95	0.0825+0.1922	0.0310*
II	10	0.0067+0.0062	
III	10	0.0087+0.0147	
LymphNode			
Metastasi			
Yes	4	0.0045+0.0048	0.6260
No	111	0.0724+0.1812	
Smoking			
Yes	13	0.0198+0.0441	0.2848
No	102	0.0758+0.1864	

NSCLC, non-small cell lung cancer, AC, adenocarcinoma; SCC, squamous cell carcinoma. *p < 0.05. The bold values provided means p value is less than 0.05.

Furthermore, smoking is currently considered the highest risk factor for NSCLC (26).To further explore whether piRNA-hsa-164586 of extracellular vesicles from serum can have predictive effects in NSCLC patients, we explored the relationship between piR-hsa-164586 and clinically relevant characteristics. The age, gender, tumor pathological subtypes, lymph node metastasis, tumor TNM staging, and smoking status of patients were all taken into consideration. As shown in **Figures S3C, D**, age and gender were not related to the expression of piRNAs (when comparing between each set of two groups). However, when three groups were compared, the expression of piR-hsa-164586 was found to be correlated with age (**Table 2**). The expression of piR-hsa-164586 showed no significant difference between the two pathological types (**Figure S3E**). Based on whether they smoked or not, we divided all of the patients into two groups; there was no difference in the expression of piRNA between these two groups (**Figure S3F**).

Lung, breast, liver, gastric, and colorectal cancers are the top five cancers related to death in the world (27, 28). Based on the above experiment, we also explored the expression of piRNAs of extracellular vesicles from serum in four other cancers with higher incidence after collecting small sample sizes for each type of cancer, including liver cancer (LC) (29), gastric cancer (GC) (30), breast cancer (BC) (31), and colorectal cancer (CRC) (32). We showed that piR-hsa-164586 was significantly overexpressed in NSCLC, LC, and GC, while there were no differences between BC, CRC, and healthy individuals (**Figure S4A**).

## Discussion

The inefficiency of existing tumor markers is the main reason for the low survival rate of cancer. This means there is an urgent need to discover new, highly sensitive, and specific markers for the early diagnosis of cancer. Exosomes are disc-shaped vesicles that can be secreted by almost all cell types. The various components they carry can be used as biomarkers for disease diagnosis (33–35). Recently, it has been increasingly shown that piRNAs exist stably in the body fluids and have the potential to become biomarkers for the diagnosis of malignant tumors (36–39). However, the diagnostic potential of piRNAs of extracellular vesicles from serum for NSCLC has not yet been reported. Hence, the current study investigated whether serum-derived piR-hsa-164586 may have the potential as a diagnostic marker for NSCLC. In this study, we first isolated extracellular vesicles from serum using a polymer precipitation kit and further identified and analyzed their surface characteristics using TEM, NTA, and Western blot. Extracellular vesicle preparations were confirmed to be positive for the endosomal marker TSG101 and other vesicle-associated proteins (such as CD9 and CD81) and negative for the non-extracellular vesicle marker calnexin (**Figure S5**). Subsequently, we screened out piR-hsa-164586 using high-throughput sequencing and then verified its expression level in cancer patients and healthy individuals by qPCR. The results of the study demonstrated that the expression level of piR-hsa-164586 in patients with NSCLC was significantly higher than that in healthy individuals. Surprisingly, we also found that piR-hsa-164586 of extracellular vesicles was increased significantly in patients with early-stage NSCLC. However, the distribution of sample size between patients with NSCLC and healthy subjects is unbalanced, and the sample size of healthy subjects is about 41% of that of patients with NSCLC, which may amplify the detection of differences, emphasizing statistical differences that are not clinically relevant. In addition, the uneven distribution of confounding factors (such as gender and age) among different subgroups of participants also causes some interference to the results, but the population included in this study is basically the same in terms of age and gender, as shown in **Table S2**. However, more confounding factors need to be excluded for further evidence. Therefore, there is still a long way to go for piR-hsa-164586 to be applied in clinical practice, and more studies are needed in the future.

Diagnostic performance is the most important indicator of tumor markers. Therefore, we further analyzed the diagnostic performance of piR-hsa-164586 of extracellular vesicles in stage I and all stages of NSCLC patients by ROC curve analysis (**Tables S3**, **S4**). We found that independent piR-hsa-164586 had a relatively good performance in differentiating patients with NSCLC from healthy individuals compared with CYFRA21-1 (0.493). The AUC of piR-has-164586 was 0.624 (sensitivity, 0.523; specificity, 0.601). Meanwhile, in the current study, piR-hsa-164586 of extracellular vesicles showed a sensitivity of 0.535 and a specificity of 0.587 in stage I NSCLC patients.

Stability is the prerequisite for the clinical application of tumor markers (40–42). First, we verified that piRNA was enriched in extracellular vesicles of serum instead of EDS. Next, we verified the stability of piRNAs in extracellular vesicles from serum. The results confirmed that the expression of piRNA did not decrease significantly even though extracellular vesicles were treated with RNase A. In addition, the expression level of piRNA in extracellular vesicles was not significantly affected by long-term storage at room temperature. These results are consistent with previous research results (43). All these results indicate that piRNAs are stable in extracellular vesicles from serum, which lays the foundation for further research.

We also compared the piRNA expression in extracellular vesicles from the serum of patients with NSCLC before and after surgery. The results showed that the expression level of piR-hsa-164586 significantly decreased after the operation, indicating that piR-hsa-164586 may have a certain predictive effect on the prognosis of surgical patients.

Finally, we correlated the piR-hsa-164586 of extracellular vesicles with the clinical characteristics of patients with NSCLC, such as age, gender, pathological subtype of cancer, lymph node metastasis, TNM staging of tumor, and smoking. Experimental results showed that the expression level of piR-hsa-164586 of extracellular vesicles from serum was related to TNM staging and age of NSCLC patients. The expression of piR-hsa-164586 was significantly upregulated in stage I without lymph node metastasis. These results indicate that piR-hsa-164586 can predict the diagnosis of patients with early stages of NSCLC.

The molecular mechanisms of piRNAs that exert function are largely unclear. A previous study has shown that piR-hsa-211106 inhibits the progression of lung adenocarcinoma and enhances chemotherapy sensitivity by pyruvate carboxylase (44). piRNAs also work by interacting with piwi proteins and recruiting methyltransferases. For instance,piR-651 could promote cell proliferation and migration and inhibit apoptosis in breast cancer by facilitating DNMT1-mediated PTEN promoter methylation (45). More recently, piRNA can participate in the occurrence and development of tumors by regulating m6A RNA methylation (46). These findings expand our understanding of the regulatory role of piRNA in cancer. We will explore the specific mechanism of piR-hsa-164586 affecting the occurrence and development of NSCLC in the next step.

Our research has certain limitations. First, the sample size was small, particularly for verifying the expression of piR-hsa-164586 in NSCLC and other cancers, so it is necessary to verify piRNAs of extracellular vesicles in a larger population. Second, we only discussed piR-hsa-164586 of extracellular vesicles as a potential biomarker for NSCLC, and the mechanisms involved have not been analyzed through functional experiments. Thus, we will perform further experimental validation to explore the potential role of piR-hsa-164586 of extracellular vesicles as a diagnostic biomarker for NSCLC and to explore the mechanisms associated with NSCLC.

## Conclusions

In this study, we discovered that piR-hsa-164586 was enriched in the extracellular vesicles of the serum of NSCLC patients. Hence, it may be a noninvasive, stable, and convenient biomarker for human NSCLC diagnosis, prognosis, and particularly prediction of postoperative outcomes. Liquid biopsy is the trend of tumor diagnosis in the future, as it can not only make up for the deficiency of the existing diagnostic methods but also provide new research ideas based on existing research. As research progresses, the diagnosis of tumors tends to become more accurate.

## Data availability statement

The original contributions presented in the study are included in the article/**Supplementary Materials**. Further inquiries can be directed to the corresponding authors.

## Ethics statement

The studies involving human participants were reviewed and approved by the Ethics Committee of Qingdao University Hospital. The patients/participants provided their written informed consent to participate in this study.

## Author contributions

YaL, WX, XY, and YD contributed to the conception of the work. JG, XH, ZW, ML, MW, and YiL participated in clinical sample collection and data collation. YaL drafted the manuscript. WX, XY, and YD reviewed and modified for the manuscript. All authors listed have made a substantial, direct, and intellectual contribution to the work and approved it for publication.

## Funding

This work was supported by the National Natural Science Foundation of China (No. 81770900) and the Key Projects of Science and Technology Benefiting People in Qingdao (No. 20-3-4-43-nsh).

## Acknowledgments

The authors thank all the team who participated in the project research.

## Conflict of interest

The authors declare that the research was conducted in the absence of any commercial or financial relationships that could be construed as a potential conflict of interest.

## Publisher’s note

All claims expressed in this article are solely those of the authors and do not necessarily represent those of their affiliated organizations, or those of the publisher, the editors and the reviewers. Any product that may be evaluated in this article, or claim that may be made by its manufacturer, is not guaranteed or endorsed by the publisher.

## Abbreviations

NSCLC, non-small cell lung cancer; piRNA, PIWI interacting RNA; qRT-PCR, quantitative real-time PCR; ROC, receiver operating characteristic; AUC, area under the curve; SC, squamous cell carcinoma; AC, adenocarcinoma; TEM, transmission electron microscope; NTA, nanoparticle tracking analysis; CYFRA21-1, cytokeratin-19-fragment; EDS, extracellular vesicles-depleted suspension; LC, liver cancer; GC, gastric cancer; BC, breast cancer; CRC, colorectal cancer; F, forward; R, reverse; SN, sensitivity; SP, specificity; SD, standard deviation; CI, confidence interval.
