# Supplementary material for: Serum-derived piR-hsa-164586 of extracellular vesicles as a novel biomarker for early diagnosis of non-small cell lung cancer
Source: Front Oncol. 2022 Sep 28;12:850363. doi: 10.3389/fonc.2022.850363 (PMC9559724; doi:10.3389/fonc.2022.850363)
Supplement: Supplementary file 1 [file DataSheet_1.docx]

Supplementary Material

**Supplementary Tables**

**Table S1.**RNA Sequencing Results of piRNA in Cancer tissue and paired paracancer tissue

| Gene Name | Log2FC | Fold Change | *P* value |
| --- | --- | --- | --- |
| piR-hsa-143056  piR-hsa-158651  piR-hsa-171929  piR-hsa-164586  piR-hsa-148318  piR-hsa-93750  piR-hsa-137463  piR-hsa-34316 | 4.895674948  2.350069403  2.024020172  1.708612102  1.550884567  1.245156584  1.23520259  1.215851886 | 29.76768138  5.098487774  4.067155561  3.268462403  2.929967308  2.370442806  2.354144032  2.322778984 | P<0.05  P<0.05  P<0.05  P<0.05  P<0.05  P<0.05  P<0.05  P<0.05 |

## Table S2.Background demographic of the study cohorts

| Paremeter | Healthy | StageⅠof NSCLC | NSCLC | Total |
| --- | --- | --- | --- | --- |
| N  Age Mean±SD  Range  Gender Male  Female | 47  47.98±9.87  30-68  13(28%)  34(72%) | 95  58.16±9.47  31-78  25(26%)  70(74%) | 115  58.58±9.41  31-81  28(24%)  87(76%) | 162  55.61±10.64  30-81  52(32%)  110(68%) |

## Table S3.**Clinical diagnosis perforance about various marker alone and their combination effects for stageⅠof NSCLC diagnosis**

| Healthy vs stageⅠof NSCLC | | | | | |
| --- | --- | --- | --- | --- | --- |
| Marker  CYRA21-1  piR-hsa-164586  CYRA21-1+piR-hsa-164586 | AUC  0.506  0.623  0.645 | Sensitivity%  0.478  0.535  0.541 | Specificity%  0.526  0.587  0.603 | *p* value  0.941  0.031  0.011 | 95% CI  0.395-0.617  0.527-0.720  0.550-0.741 |

## Table S4.**Clinical diagnosis perforance about various marker alone and their combination effects for all stages of NSCLC diagnosis**

| Healthy vs all stages of NSCLC | | | | | |
| --- | --- | --- | --- | --- | --- |
| Marker CYFRA21-1  piR-hsa-164586  CYFRA21-1+piR-hsa-164586 | AUC 0.493  0.624  0.642 | Sensitivity% 0.469  0.523  0.535 | Specificity% 0.523  0.601  0.606 | *p* value 0.897  0.026  0.011 | 95%CI 0.385-0.600  0.530-0.718  0.550-0.734 |

## Supplementary Figures

B

A


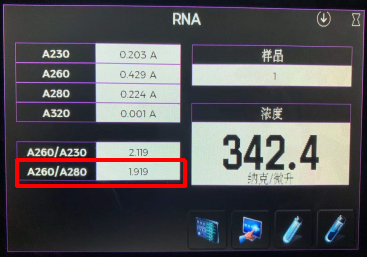

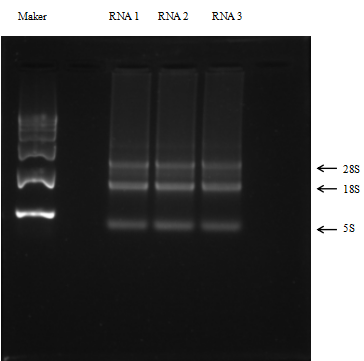


**Figure S1**.Verification the purity and quality of the isolated RNA.(A)OD260/280;(B)agarose gel electrophoresis experiments.

A

D

C

B

F

G

E

I

H

**Figure S2.** Screening of differentially expressed piRNAs with small serum samples.(A)The eight most distinctive piRNAs screened out by tissue sequencing(B)piR-hsa-164586.(C)piR-hsa-93750.(D)piR-hsa-137463.(E)piR-hsa-158651.(F)piR-hsa-34316.(G)piR-hsa-147056.(H)piR-hsa-148318.(I)piR-hsa-171929.

B

A

D

C

E

F

**Figure S3.**Correlation between piRNA level and clinical characteristics of NSCLC patients..Expression levels of piR-hsa-164586 with(A)TNM stage,(B)lymph node metastasis,(C)age,(D)gender,(E)main pathological types,(F)Smoking .(ns,no significance；*p＜0.05,**p＜0.01,***p＜0.001)

A

**Figure S4.**The expression of piRNA in different types of cancer.(A)piR-hsa-164586(LC,live cancer;GC,gastric cancer;BC,breast cancer;CRC,colorectal cancer)

D

C

B

A

Calnexin

CD81

TSG101

CD9


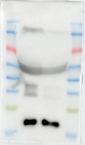

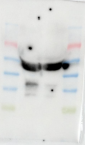

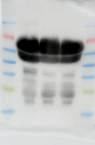

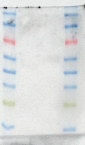


25KD

20KD

40KD

50KD

25KD

20KD

**Figure S5.S**pecific marker proteins (CD9,TSG101and CD81) and negative marker(Calnexin)of extracellular vesiclewere detected by Western blotting.(A)CD9,(B)TSG101,(C)CD81,(D)Calnexin
